# Low Docosahexaenoic Acid, Dihomo-Gamma-Linolenic Acid, and Arachidonic Acid Levels Associated with Long-Term Mortality in Patients with Acute Decompensated Heart Failure in Different Nutritional Statuses

**DOI:** 10.3390/nu9090956

**Published:** 2017-08-30

**Authors:** Shohei Ouchi, Tetsuro Miyazaki, Kazunori Shimada, Yurina Sugita, Megumi Shimizu, Azusa Murata, Takao Kato, Tatsuro Aikawa, Shoko Suda, Tomoyuki Shiozawa, Masaru Hiki, Shuhei Takahashi, Hiroshi Iwata, Takatoshi Kasai, Katsumi Miyauchi, Hiroyuki Daida

**Affiliations:** Department of Cardiovascular Medicine, Juntendo University School of Medicine, 2-1-1 Hongo Bunkyo-ku, Tokyo 113-8421, Japan; uchi@juntendo.ac.jp (S.O.); shimakaz@juntendo.ac.jp (K.S.); yrsugita@juntendo.ac.jp (Y.S.); megumi-s@juntendo.ac.jp (M.S.); azmurata@juntendo.ac.jp (A.M.); tkatou@juntendo.ac.jp (T.K.); taikawa@juntendo.ac.jp (T.A.); ssuda@juntendo.ac.jp (S.S.); t-shio@juntendo.ac.jp (T.S.); ma-hiki@juntendo.ac.jp (M.H.); syutaka@juntendo.ac.jp (S.T.); h-iwata@juntendo.ac.jp (H.I.); tkasai@juntendo.ac.jp (T.K.); ktmmy@juntendo.ac.jp (K.M.); daida@juntendo.ac.jp (H.D.)

**Keywords:** polyunsaturated fatty acids (PUFAs), eicosapentaenoic acid (EPA), docosahexaenoic acid (DHA), dihomo-gamma-linolenic acid (DGLA), arachidonic acid (AA), geriatric nutritional risk index (GNRI), acute decompensated heart failure (ADHF), nutritional status

## Abstract

The clinical significance of polyunsaturated fatty acids (PUFAs) in acute decompensated heart failure (ADHF) in various nutritional statuses remains unclear. For this study, we enrolled 267 patients with ADHF admitted to the cardiac intensive care unit at Juntendo University hospital between April 2012 and March 2014. The association between long-term mortality, the geriatric nutritional risk index (GNRI), and levels of PUFAs, including eicosapentaenoic acid (EPA), docosahexaenoic acid (DHA), dihomo-gamma-linolenic acid (DGLA), and arachidonic acid (AA) was investigated. The median age was 73 (64–82) years, and mortality was 29% (62 patients). The event-free survival rates for all-cause death were higher in patients with high PUFA levels or GNRI than in those with low PUFA levels or GNRI (*p* < 0.05 for all). In particular, high DGLA in the low-GNRI group and high DHA or AA in the high-GNRI group were associated with high event-free survival (*p* < 0.05 for all). After accounting for confounding variables, DHA, DGLA, and AA, but not EPA, were associated with long-term mortality (*p* < 0.01 for all). This study concludes that in patients with ADHF, decreased levels of DHA, DGLA, and AA are independently associated with long-term mortality in the various nutritional statuses.

## 1. Introduction

Malnutrition is highly prevalent in patients with heart failure, particularly in those with advanced heart failure and acute decompensated heart failure (ADHF) [1,2]. Moreover, undernutrition is associated with unfavorable prognosis and mortality in patients with heart failure [3,4,5]. The geriatric nutritional risk index (GNRI) is calculated from the serum albumin level and body mass index and is widely used for evaluating nutritional status [6,7,8,9]. Previous studies have reported that low GNRI levels are significantly associated with poor prognosis in patients with heart failure. In particular, in elderly patients, low GNRI levels are predictive of morbidity and mortality [3,6,10,11]. In addition, frailty has been shown to be an independent predictor of early disability, long-term mortality, and readmission in patients with heart failure [12,13]. However, it remains difficult to improve the nutritional status and physical ability in these patients despite treatment [14,15].

Polyunsaturated fatty acids (PUFAs) are characterized by the presence of at least two carbon–carbon double bonds. Omega-3 PUFAs, including eicosapentaenoic acid (EPA) and docosahexaenoic acid (DHA), have the first double bond at the third carbon from the methyl terminus, and omega-6 PUFAs, including arachidonic acid (AA) and dihomo-gamma-linolenic acid (DGLA), have the first bond at the sixth carbon from the methyl terminus [16,17]. Fish oils are rich in omega-3 PUFAs, whereas sunflower, safflower, and corn oils and farm animal meat are rich in omega-6 PUFAs [18,19,20]. Omega-3 PUFAs play an important role in preventing cardiovascular diseases [21,22,23,24,25,26], and omega-6 PUFAs are associated with long-term mortality in patients with ADHF [27]. The clinical significance of PUFAs in various nutritional statuses remains unclear. Therefore, we investigated the association of PUFAs with the prognosis of patients with ADHF in different grades of GNRI.

## 2. Materials and Methods

### 2.1. Patients

The present study was part of an ongoing cohort study of biomarkers in patients admitted to the cardiac intensive care unit (UMIN-CTR; UMIN000007555), in which the PUFA hypothesis was generated retrospectively; however, data were collected systematically and prospectively. In total, 267 consecutive patients with ADHF admitted to the cardiac intensive care unit at Juntendo University Hospital between April 2012 and March 2014 were enrolled in the present study. Patients with end-stage kidney disease (defined as an estimated glomerular filtration rate of <15 mL/min/1.73 m^2^), those with malignancy, and those receiving omega-3 therapy were excluded from the study. Also, alcoholics or drug abusers were excluded.

ADHF was defined according to the diagnostic criteria of the Framingham study [28]. Diabetes mellitus was defined as a previous diagnosis from medical records, a hemoglobin A1c (national glycol-hemoglobin standardization program calculation) level of ≥6.5%, or treatment with oral antidiabetic agents or insulin. Dyslipidemia was defined as a previous diagnosis from medical records, abnormal lipid profiles (i.e., triglyceride level ≥150 mg/dL, low-density lipoprotein cholesterol level ≥140 mg/dL, or high-density lipoprotein cholesterol level ≤40 mg/dL), or treatment with antidyslipidemic agents. Hypertension was defined as a previous diagnosis, which was defined as having a systolic blood pressure of ≥140 mmHg and/or diastolic blood pressure of ≥90 mmHg [29] in the medical records or treatment with antihypertensive agents. All subjects gave their informed consent for inclusion before they participated in the study. The study was conducted in accordance with the Declaration of Helsinki, and the protocol was approved by the Ethics Committee of Juntendo University Hospital (project identification code: 871).

### 2.2. Blood Sampling

Whole blood samples were drawn after an overnight fast, within 24 h of admission. Serum levels of EPA, DHA, DGLA, and AA were measured by SRL Inc. (Tokyo, Japan) using gas chromatography [30]. Plasma levels of total cholesterol, and creatinine were measured using enzymatic methods, triglyceride levels using visible absorption spectrometry, high-density lipoprotein cholesterol levels using the direct method, and low-density lipoprotein cholesterol levels were calculated using the Friedewald formula. Hemoglobin A1c levels were measured using high-performance liquid chromatography, total protein levels using the Biuret method, albumin levels using the bromocresol purple method, cholinesterase levels using a modified Japan Society of Clinical Chemistry reference method, and brain natriuretic peptide levels using the one-step sandwich enzyme immunoassay method. The estimated glomerular filtration rate was calculated on the basis of the Japanese equation using the serum creatinine level, age, and gender as follows: estimated glomerular filtration rate (mL/min/1.73 m^2^) = 194 × creatinine^−1.094^ × age^−0.287^ (for females: × 0.739) [31]. GNRI was calculated from serum albumin level and body weight and height obtained on admission as follows: GNRI = 14.89 × serum albumin (g/dL) + 41.7 × (actual body weight/ideal body weight). Actual body weight/ideal body weight was set to 1 when the patient’s body weight exceeded the ideal body weight. The ideal body weight in the present study was calculated using a body mass index of 22 kg/m^2^. From these GNRI scores, four grades of nutrition-related risk were identified: no risk (>98), low risk (92 to ≤98), moderate risk (82 to <92), and high risk (<82) [6,32].

### 2.3. Statistical Analysis

Continuous variables are expressed as medians with an interquartile range, and categorical variables are expressed as number and percentages. Comparisons of continuous variables were performed using the Student’s *t*-test or Mann-Whitney U test. Categorical variables were analyzed using the chi-square test or Fisher’s exact probability test.

The primary endpoint was all-cause death, and the patients were followed up until December 2015. The patients were categorized according to survival into the survivor or nonsurvivor group. Unadjusted cumulative event rates for the primary endpoint were estimated using the Kaplan-Meier method, and they were compared between groups using the log-rank test. Cutoffs were defined using median PUFA levels. The cutoff value of GNRI was set to 92. Univariate and multivariate Cox regression analyses were performed to identify the predictors of the primary endpoint. Hazard ratios (HRs) and 95% confidence intervals (CIs) were also calculated. Age, gender, body mass index, diabetes mellitus, dyslipidemia, hypertension, smoking, renal function (serum creatinine level), cardiac function (left ventricular ejection fraction), and GNRI were included in multivariate analyses. JMP12 (for Windows, SAS Institute, Cary, NC, USA) was used for statistical analyses, and *p* values of <0.05 were considered statistically significant.

## 3. Results

### 3.1. Baseline Characteristics

Of the 267 patients, 214 patients were examined in the present study. Patient demographics are presented in Table 1. The median patient age was 73 (64–82) years, and 115 patients (53.7%) were males. The survivor group included 152 patients (71.0%), and the nonsurvivor group included 62 patients (29.0%). The mean follow-up duration was 22.6 ± 14.2 months, and the maximum follow-up duration was 45.6 months. The patients in the nonsurvivor group were significantly older and had a lower body mass index, systolic blood pressure, diastolic blood pressure, heart rate, and prevalence of dyslipidemia than those in the survivor group. Furthermore, the patients in the nonsurvivor group had significantly lower levels of total cholesterol, low-density lipoprotein cholesterol, albumin, and cholinesterase and significantly higher levels of creatinine and brain natriuretic peptide than those in the survivor group. The administration of diuretic, antiplatelet, and inotropic agents was more common in the nonsurvivor group than in the survivor group (Table 1).

### 3.2. PUFA Levels and GNRI in the Survivor and Nonsurvivor Groups

PUFA levels were significantly lower in the nonsurvivor group than in the survivor group: EPA, 34.4 (26.1–50.9) vs. 42.6 (31.2–62.6) μg/mL, *p* = 0.04; DHA, 104.3 (81.1–128.2) vs. 112.4 (93.9–140.6) μg/mL, *p* = 0.04; DGLA, 19.1 (14.7–22.9) vs. 23.0 (18.5–32.0) μg/mL, *p* < 0.01; AA, 137.2 (107.2–158.1) vs. 160.2 (122.6–190.2) μg/mL, *p* = 0.01. The GNRI was also significantly lower in the nonsurvivor group than in the survivor group: 87.9 (81.4–90.2) vs. 92.3 (85.6–96.6) μg/mL, *p* < 0.01 (Table 1).

### 3.3. Cumulative Event-Free Survival Rates Based on PUFA Levels and GNRI

**Figure 1:**
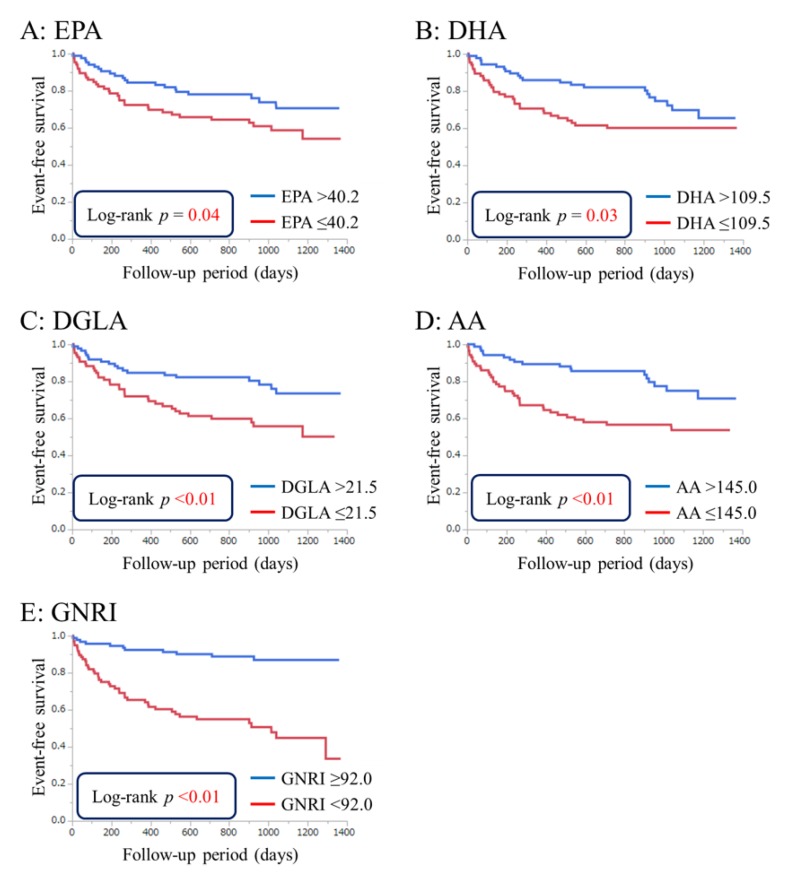
Event-free survival curves for all-cause death in patients with acute decompensated heart failure. Unadjusted cumulative event rates for the primary endpoint (all-cause death) were estimated using the Kaplan-Meier method and compared between the groups using the log-rank test. Cutoff values were defined as the median polyunsaturated fatty acid levels. (**A**) eicosapentaenoic acid (EPA), 40.2 μg/mL; (**B**) docosahexaenoic acid (DHA), 109.5 μg/mL; (**C**) dihomo-gamma-linolenic acid (DGLA), 21.5 μg/mL; (**D**) arachidonic acid (AA), 145.0 μg/mL; (**E**) The patients were also divided into high-GNRI (no or low risk; GNRI ≥92.0) and low-GNRI (moderate or high risk; GNRI <92.0) groups.

Kaplan-Meier curves were constructed to demonstrate the unadjusted event-free rate for all-cause death. The patients were categorized into two groups based on median PUFA levels (EPA, 40.2 μg/mL; DHA, 109.5 μg/mL; DGLA, 21.5 μg/mL; and AA, 145.0 μg/mL). The patients were also divided into high-GNRI (no or low risk (GNRI ≥92.0); *n* = 84) and low-GNRI (moderate or high risk (GNRI <92.0); *n* = 112) groups. Event-free survival rates were higher in the high-PUFA or high-GNRI groups than in the low-PUFA or low-GNRI groups (EPA, *p* = 0.04; DHA, *p* = 0.03; DGLA, AA, and GNRI, *p* < 0.01) (Figure 1).

### 3.4. Effects of PUFA Levels on Event-Free Survival Independent of GNRI

**Figure 2:**
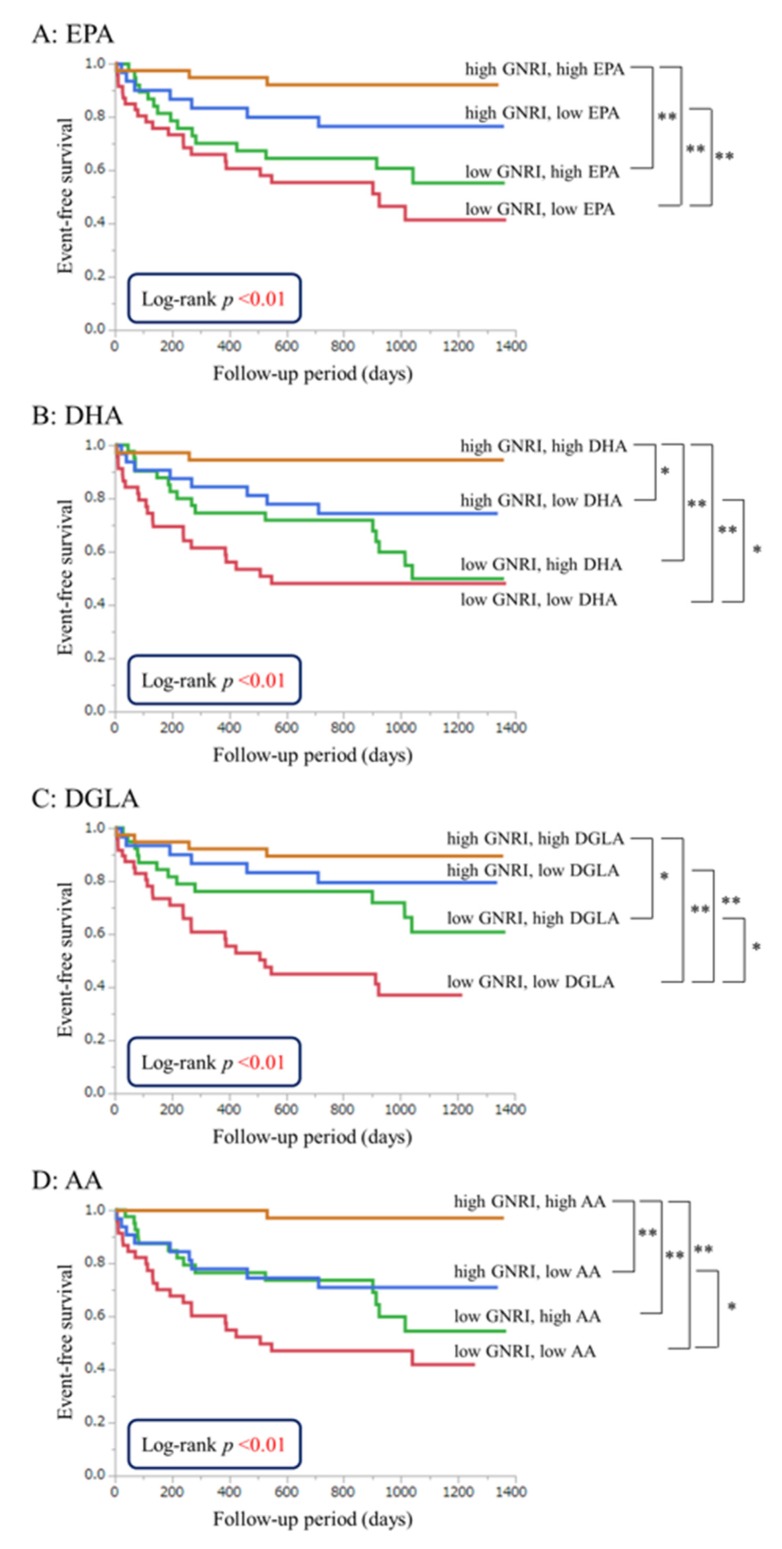
Event-free survival curves for all-cause death in patients with acute decompensated heart failure (ADHF). Unadjusted cumulative event rates for the primary endpoint (all-cause death) were estimated using the Kaplan-Meier method and compared between groups using the log-rank test. The patients were categorized into four groups on the basis of the median PUFA levels in the high-GNRI (no or low risk; GNRI ≥92.0) and low-GNRI (moderate or high risk; GNRI <92.0) groups. (**A**) eicosapentaenoic acid (EPA), 40.2 μg/mL; (**B**) docosahexaenoic acid (DHA), 109.5 μg/mL; (**C**) dihomo-gamma-linolenic acid (DGLA), 21.5 μg/mL; and (**D**) arachidonic acid (AA), 145.0 μg/mL. * *p* < 0.05; ** *p* < 0.01.

The patients were categorized into four groups on the basis of median PUFA levels in the high- and low-GNRI groups (EPA, 40.2 μg/mL; DHA, 109.5 μg/mL; DGLA, 21.5 μg/mL; AA, 145.0 μg/mL; and GNRI, 92.0). Kaplan-Meier curves for all-cause death and comparisons of the risk of all-cause death in each group are shown in Figure 2. The low-GNRI and high-DGLA groups had a significantly higher event-free survival risk than those in the low-GNRI and low-DGLA groups (HR, 0.43; CI, 0.21–0.84; *p* = 0.01). Low DGLA levels were associated with poor prognosis in patients with a poor nutritional status. The high-GNRI and high-DHA groups had a significantly higher event-free survival risk than those in the high-GNRI and low-DHA groups (HR, 0.20; CI, 0.03–0.78; *p* = 0.02). The high-GNRI and high-AA groups had a significantly higher event-free survival risk than those in the high-GNRI and low-AA groups (HR, 0.08; CI, 0.01–0.42; *p* < 0.01). Low DHA and AA levels were associated with poor prognosis in patients with a good nutritional status.

### 3.5. Univariate and Multivariate Analyses of Parameters Contributing to Long-Term Mortality

Univariate Cox regression analyses revealed that age, body mass index, dyslipidemia, smoking, creatinine, GNRI, DHA, DGLA, and AA were associated with long-term mortality, while EPA was not. After accounting for confounding variables, DHA (10 μg/mL increase; HR, 0.87; 95% CI, 0.78–0.96; and *p* < 0.01), DGLA (10 μg/mL increase; HR, 0.54; 95% CI, 0.34–0.80; and *p* < 0.01), and AA (10 μg/mL increase, HR, 0.92; 95% CI, 0.86–0.98; and *p* < 0.01) were associated with long-term mortality, but EPA was not (Table 2).

## 4. Discussion

The present study demonstrated that low serum PUFA levels in patients with ADHF were associated with poor long-term prognosis in the different grades of GNRI. Decreased levels of DHA, DGLA, and AA, but not EPA, were independently associated with long-term mortality in patients with ADHF in the various nutritional statuses, suggesting the importance of evaluating the nutritional status using fatty acid metabolism in addition to the evaluation using protein metabolism, similar to GNRI.

In this study population, patients with low PUFA levels on admission showed about 40% to 50% of mortality during the follow-up period of 22.6 ± 14.2 months. These low PUFA levels may reflect the worse clinical status of patients on admission, which induces these worse outcomes. The shortage of these essential fatty acids, especially DHA, DGLA, and AA, may be caused by poor dietary intake and/or exhaustion of nutrients from heart failure. These results suggest the importance of nutritional management, including intake of essential fatty acids, for patients who have risks of ADHF.

Omega-3 PUFAs, including EPA and DHA, have anti-inflammatory, antiatherogenic, and antiarrhythmic effects [20,26,33]. They synthesize series 3 prostaglandins, which reduce inflammation, leading to a decrease in the rate of formation of series 2 prostaglandins, which increase inflammation and induce platelet aggregation and vasoconstriction [20,33,34,35,36]. These beneficial effects of omega-3 PUFAs may contribute to improved prognosis in patients with ADHF.

Interpretation of the results of omega-6 PUFAs studies is complicated. AA is converted to series 2 prostaglandins, which are relatively harmful; low levels of series 2 prostaglandins cause difficulty in maintaining hemostasis, and excess levels increase inflammation [33,37,38]. DGLA, another omega-6 PUFA, is converted to series 1 prostaglandins, which have beneficial effects, including aggregation of platelets, reduction of inflammation, and maintenance of homeostasis [39,40,41]. Further research is warranted to clarify the role of omega-6 PUFAs, particularly AA, in the pathogenesis of ADHF.

Both low GNRI and serum PUFA levels are indicators of malnutrition, which is highly prevalent in patients with heart failure, particularly in those with advanced heart failure and ADHF. Malnutrition is significantly associated with unfavorable prognosis and high mortality in patients with heart failure [1]. Accelerated catabolism reduces proteins, including albumin and muscle mass, which exacerbates frailty in severely ill patients. Malnutrition and frailty are well-known predictors for poor prognosis, but their treatment remains challenging [1,13]. Although several studies have reported trials of exercise and nutritional supplementation for patients with heart failure, to our knowledge, no study has demonstrated significant improvement in prognosis [42,43,44,45]. The present study demonstrated that decreased DGLA levels are significantly associated with long-term mortality in patients with a poor nutritional status. In addition, decreasing the activity of delta-5 desaturase, which converts DGLA to AA in the omega-6 pathway, increases DGLA levels [46]. These results suggest that both supplementation of DGLA and delta-5 desaturase inhibitors is useful in the treatment of patients with malnutrition and heart failure [47].

Decreased DHA levels, but not EPA, were independently associated with long-term mortality in patients with ADHF. PUFAs play an important role in cellular membrane function, and their levels vary in cell membranes of different tissues [33]. DHA is abundant in the cell membrane of cardiocytes [48,49], but EPA is scarce. This difference in cardiocyte cell membrane composition may contribute to the distinct effects of DHA and EPA in patients with ADHF.

GNRI, an established indicator of nutritional status, is calculated using the serum albumin level and body mass index. It is also considered an indicator of protein metabolism but not lipid and fatty acid metabolisms [50]. In this study, patients with ADHF who had low GNRI and PUFA levels had high mortality, suggesting the importance of not only protein catabolism but also fatty acid catabolism in evaluating nutritional status.

This study has several limitations. First, the study was conducted in a single institution and the study population was relatively small. Studies with a larger sample size and multicenter trials are needed to evaluate the association between PUFAs and prognosis in patients with ADHF. Second, serum albumin levels in patients with ADHF may have been affected by body fluid volume because blood samples were drawn during the acute phase. Therefore, GNRI in the present study may be lower than that during the chronic phase. Third, data on the dietary status and physical activity of the patients could not be collected. Thus, we could not assess the association of PUFA levels with dietary status and physical activity before admission.

## 5. Conclusions

Both omega-3 and omega-6 PUFAs are associated with long-term mortality in patients with ADHF. Moreover, decreased levels of DHA, DGLA, and AA, but not EPA, are independently associated with long-term mortality in patients with ADHF in the various nutritional statuses. In particular, a low DGLA level is predictive of long-term mortality in patients with ADHF and a poor nutritional status, and low DHA and AA levels are predictive of long-term mortality in patients with ADHF and a good nutritional status. Therefore, further studies are warranted to clarify the effective supplementation of omega-3 and omega-6 PUFAs for patients with ADHF with different nutritional statuses. The results also suggest the importance of evaluating nutritional status using fatty acid metabolism, in addition to protein metabolism.

## Figures and Tables

**Table 1:** Characteristics of the study subject.

	All (*n* = 214)	Survivor Group (*n* = 152)	Nonsurvivor Group (*n* = 62)	*p*-Value *
Age (years)	73 (64–82)	72 (61–81)	74 (69–82)	0.03
Male (*n*, %)	115 (53.7)	77 (50.7)	38 (61.3)	NS
Body mass index (kg/m^2^)	22.9 (20.4–25.7)	23.4 (21.1–26.7)	21.1 (19.5–23.4)	<0.01
Systolic blood pressure (mmHg)	130 (110–149)	135 (115–155)	115 (94–135)	<0.01
Diastolic blood pressure (mmHg)	77 (62–90)	80 (68–96)	65 (56–77)	<0.01
Heart rate (per min)	93 (74–112)	97 (75–118)	87 (71–101)	0.01
Left ventricular ejection fraction (%)	35 (25–49)	38 (26–51)	32 (20–45)	NS
Diabetes mellitus (*n*, %)	89 (41.6)	61 (40.1)	28 (45.2)	NS
Dyslipidemia (*n*, %)	143 (66.8)	111 (73.0)	32 (51.6)	<0.01
Hypertension (*n*, %)	142 (66.4)	100 (65.8)	42 (67.7)	NS
Smoking (current smoker) (*n*, %)	25 (11.7)	22 (14.6)	3 (4.8)	NS
Atrial fibrillation (*n*, %)	91 (42.5)	67 (44.1)	24 (38.7)	NS
Ischemic heart disease (*n*, %)	85 (39.7)	56 (36.8)	29 (46.8)	NS
Laboratory data				
Total cholesterol (mg/dL)	153 (128–178)	156 (132–186)	140 (116–161)	<0.01
Triglycerides (mg/dL)	77 (56–108)	80 (58–109)	72 (49–105)	NS
HDL-C (mg/dL)	38 (33–49)	38 (33–50)	38 (32–46)	NS
LDL-C (mg/dL)	92 (75–113)	100 (79–117)	82 (69–105)	<0.01
Creatinine (mg/dL)	0.94 (0.72–1.31)	0.87 (0.67–1.22)	1.25 (0.94–1.80)	<0.01
HbA1c (%)	6.0 (5.5–6.7)	6.0 (5.5–6.8)	6.0 (5.6–6.6)	NS
Total protein (g/dL)	6.4 (6.0–6.7)	6.4 (6.1–6.7)	6.3 (5.9–6.9)	NS
Albumin (g/dL)	3.4 (3.0–3.6)	3.5 (3.1–3.8)	3.2 (2.9–3.4)	<0.01
Cholinesterase (U/L)	183 (138–244)	210 (154–264)	158 (105–183)	<0.01
Brain natriuretic peptide (pg/mL)	800 (445–1610)	627 (372–1206)	1495 (660–2246)	<0.01
EPA (μg/mL)	40.6 (28.7–61.6)	42.6 (31.2–62.6)	34.4 (26.1–50.9)	0.04
DHA (μg/mL)	109.6 (88.8–138.2)	112.4 (93.9–140.6)	104.3 (81.1–128.2)	0.04
DGLA (μg/mL)	21.8 (17.4–29.5)	23.0 (18.5–32.0)	19.1 (14.7–22.9)	<0.01
AA (μg/mL)	145.7 (117.0–182.4)	160.2 (122.6–190.2)	137.2 (107.2–158.1)	<0.01
GNRI	90.8 (84.5–94.8)	92.3 (85.6–96.6)	87.9 (81.4–90.2)	<0.01
Medication				
Diuretics (*n*, %)	115 (54.5)	66 (44.0)	49 (80.3)	<0.01
Antiplatelets (*n*, %)	77 (36.5)	44 (29.3)	33 (54.1)	<0.01
Anticoagulants (*n*, %)	75 (35.5)	47 (31.3)	28 (45.9)	NS
ACE-I/ARBs (*n*, %)	107 (50.7)	72 (48.0)	35 (57.4)	NS
β-blockers (*n*, %)	96 (45.5)	62 (41.3)	34 (55.7)	NS
Calcium channel blockers (*n*, %)	59 (28.0)	40 (26.7)	19 (31.2)	NS
Inotropic agents (*n*, %)	34 (16.1)	16 (10.7)	18 (29.5)	<0.01
Statins (*n*, %)	52 (24.6)	37 (24.7)	15 (24.6)	NS
Oral hypoglycemic agents (*n*, %)	43 (20.3)	32 (21.2)	11 (18.0)	NS
Insulin (*n*, %)	23 (10.8)	18 (11.9)	5 (8.2)	NS

Data are presented as median (interquartile range) or number (percentage). HDL-C: high-density lipoprotein cholesterol; LDL-C: low-density lipoprotein cholesterol; HbA1c: hemoglobin A1c; EPA: eicosapentaenoic acid; DHA: docosahexaenoic acid; DGLA: dihomo-gamma-linolenic acid; AA: arachidonic acid; GNRI: geriatric nutritional risk index; ACE-I: angiotensin converting enzyme inhibitor; ARB: angiotensin-2 receptor blocker; NS: not significant. * Comparisons between the survivor and the non-survivor groups.

**Table 2:** Univariate and multivariate Cox regression analyses for all-cause death.

	Univariate			Multivariate (EPA)			Multivariate (DHA)			Multivariate (DGLA)			Multivariate (AA)		
	HR	95% CI	*p*	HR	95% CI	*P*	HR	95% CI	*p*	HR	95% CI	*p*	HR	95% CI	*p*
Age, 1 year increase	1.03	1.01–1.06	<0.01	1.04	1.01–1.07	0.01	1.05	1.02–1.09	<0.01	1.04	1.00–1.07	0.03	1.04	1.01–1.08	<0.01
Male	1.42	0.86–2.39	NS	0.88	0.44–1.74	NS	0.74	0.37–1.44	NS	0.85	0.44–1.66	NS	0.83	0.42–1.64	NS
Body mass index, 1 kg/m^2^ increase	0.87	0.81–0.93	<0.01	0.95	0.87–1.03	NS	0.96	0.87–1.04	NS	0.95	0.86–1.03	NS	0.94	0.86–1.03	NS
Diabetes mellitus	1.13	0.68–1.86	NS	0.87	0.43–1.71	NS	0.87	0.44–1.72	NS	0.72	0.35–1.46	NS	0.85	0.42–1.68	NS
Dyslipidemia	0.43	0.26–0.71	<0.01	0.39	0.20–0.76	<0.01	0.43	0.22–0.82	0.01	0.51	0.26–0.98	0.04	0.46	0.24–0.89	0.02
Hypertension	1.08	0.64–1.88	NS	0.78	0.41–1.54	NS	0.84	0.44–1.70	NS	0.76	0.39–1.53	NS	0.68	0.35–1.36	NS
Smoking (current smoker)	0.31	0.08–0.84	0.02	0.49	0.09–1.82	NS	0.44	0.07–1.78	NS	0.40	0.07–1.64	NS	0.38	0.07–1.47	NS
Creatinine, 0.1 mg/dL increase	1.09	1.06–1.13	<0.01	1.07	1.02–1.12	<0.01	1.09	1.04–1.14	<0.01	1.10	1.05–1.16	<0.01	1.11	1.05–1.17	<0.01
LVEF, 1% increase	0.99	0.98–1.01	NS	1.00	0.98–1.02	NS	0.99	0.97–1.01	NS	0.99	0.98–1.01	NS	0.99	0.97–1.01	NS
GNRI, increase by 1	0.94	0.92–0.97	<0.01	0.95	0.91–0.99	0.02	0.94	0.91–0.98	<0.01	0.94	0.90–0.98	<0.01	0.94	0.91–0.98	<0.01
EPA, 10 μg/mL increase	0.90	0.79–1.01	NS	0.88	0.73–1.03	NS									
DHA, 10 μg/mL increase	0.91	0.84–0.99	0.02				0.87	0.78–0.96	<0.01						
DGLA, 10 μg/mL increase	0.55	0.37–0.76	<0.01							0.54	0.34–0.80	<0.01			
AA, 10 μg/mL increase	0.91	0.85–0.96	<0.01										0.92	0.86–0.98	<0.01

LVEF: left ventricular ejection fraction; GNRI: geriatric nutritional risk index; EPA: eicosapentaenoic acid; DHA: docosahexaenoic acid; DGLA: dihomo-gamma-linolenic acid; AA: arachidonic acid; NS = not significant.
